# Large pineal parenchymal tumor of intermediate differentiation causing compression with resultant obstructive hydrocephalus: a case report

**DOI:** 10.1097/MS9.0000000000000147

**Published:** 2023-02-08

**Authors:** Oadi N. Shrateh, Afnan W.M. Jobran, Haneen Owienah, Thaer Sweileh, Mohand Abulihya, Nadeem Shahin, Yazan Atawnah, Abdalwahab Kharousha, Hadi Dababseh, Sami Hussein

**Affiliations:** a Faculty of Medicine, Al-Quds University, Abu-Dis, Jerusalem; b Radiology Department; c Pathology Department; d Neurosurgery Department, Al-Istishari Arab Hospital, Ramallah, West Bank, Palestine; e Neuropathology Department, Hannover Medical School, Hannover, Germany

**Keywords:** brain neoplasms, case report, compression, hydrocephalus, pineal gland, pineal parenchymal tumor

## Abstract

**Introduction:**

The epithalamus region contains the tiny, functionally endocrine pineal gland, which has the shape of a pinecone. Less than 1% of adult primary intracranial malignancies are pineal parenchymal tumors, which are incredibly uncommon brain tumors. A rare variety of pineal parenchymal tumors are those with intermediate differentiation. These tumors, whose namesake refers to a malignant pineal parenchymal tumor, are intermediate between pineoblastomas and pineocytomas (a benign pineal parenchymal tumor).

**Case Presentation:**

A female patient, age 13, who had been experiencing terrible headaches on and off for a month, went to the emergency room. Along with the headache, she experienced nausea, vomiting, dizziness, and blurred eyesight. A nonenhanced computed tomography scan was used for the initial brain neuroimaging, which showed a hypodense mass posterior to the midbrain and superior to the cerebellum. A heterogeneous bulk was visible on MRI.

**Clinical Outcome:**

The headache, vertigo, visual disturbance, nausea, and vomiting have all improved, according to the patient. Both postoperative MRIs with and without contrast revealed the resolution of the obstructive hydrocephalus and the absence of any residual enhancing mass. The patient was followed up for 2 months without any complications or adverse events.

**Conclusion:**

One should carefully investigate a headache as the early symptom of many illnesses and rule out any other potential causes. This would therefore enable us to create a management structure for such a very unusual malignancy.

HIGHLIGHTS

- Pineal parenchymal tumors are rare and account for less than 1% of all central nervous system neoplasms.
- Patients with pineal parenchymal tumors of intermediate differentiation may present with headache and diplopia.
- The pineal parenchymal tumors of intermediate differentiation may be large enough to compress surrounding structures such as the cerebral aqueduct, resulting in hydrocephalus with or without signs of elevated intracranial pressure such as ataxia.

## Introduction

Pineal tumors account for 0.5% of all central nervous system (CNS) tumors in adults, 1% in young adults (aged 20–34 years), and 2.7% in children (aged 1–12 years)^1^. The 2007 World Health Organization (WHO) recategorized pineal parenchymal tumors (PPTs) from two subtypes: pineocytoma (grade I) and pineoblastoma (grade IV), into four entities, including a category of tumors located between pineocytoma and pineoblastoma in histological grade termed as PPTs of intermediate differentiation (PPTIDs) grade II/III^2^. Because of the limited number of reported cases, the classification of PPTs, particularly PPTIDs, remains debatable^3^,^4^. The exact clinical behavior of PPTIDs is not well understood, and the optimal therapeutic option for these tumors has not yet been determined^5^,^6^. However, patients with PPTIDs may present with signs of lesion-like effects, including headache and diplopia. Parinaud’s syndrome (upward gaze palsy as a result of tectal plate compression) can also be experienced^7^. Herein, we report a 13-year-old female patient diagnosed with a large PPTID obstructing the ventricular system with resultant obstructive hydrocephalus. This case report has been reported in line with the Surgical CAse REport (SCARE) criteria^8^.

## Case presentation

**Figure 1:**
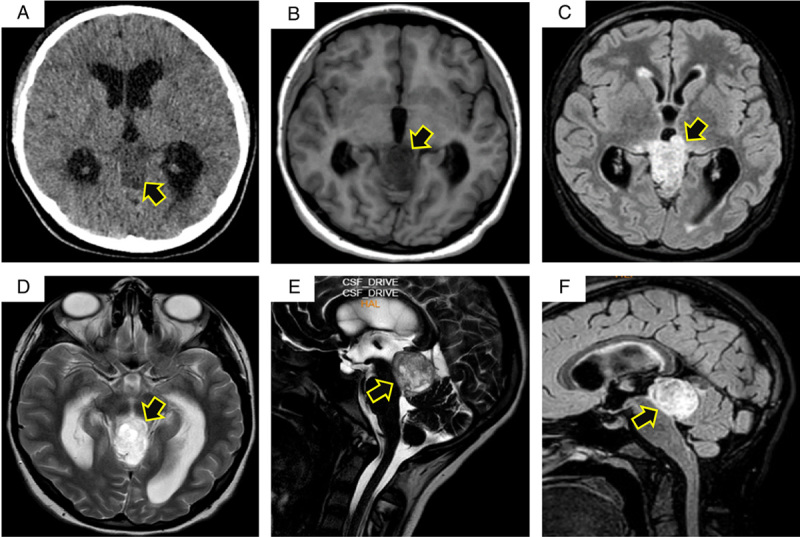
(A) Axial nonenhanced computed tomography scan showing a hypodense mass; (B) T1 sequence axial MRI; (C) FLAIR (fluid-attenuated inversion recovery) sequence axial MRI; (D) T2 sequence axial MRI; (E) FIESTA (fast imaging employing steady-state acquisition) sequence sagittal MRI; (F) FLAIR sequence sagittal MRI. CSF, cerebrospinal fluid.

**Figure 2:**
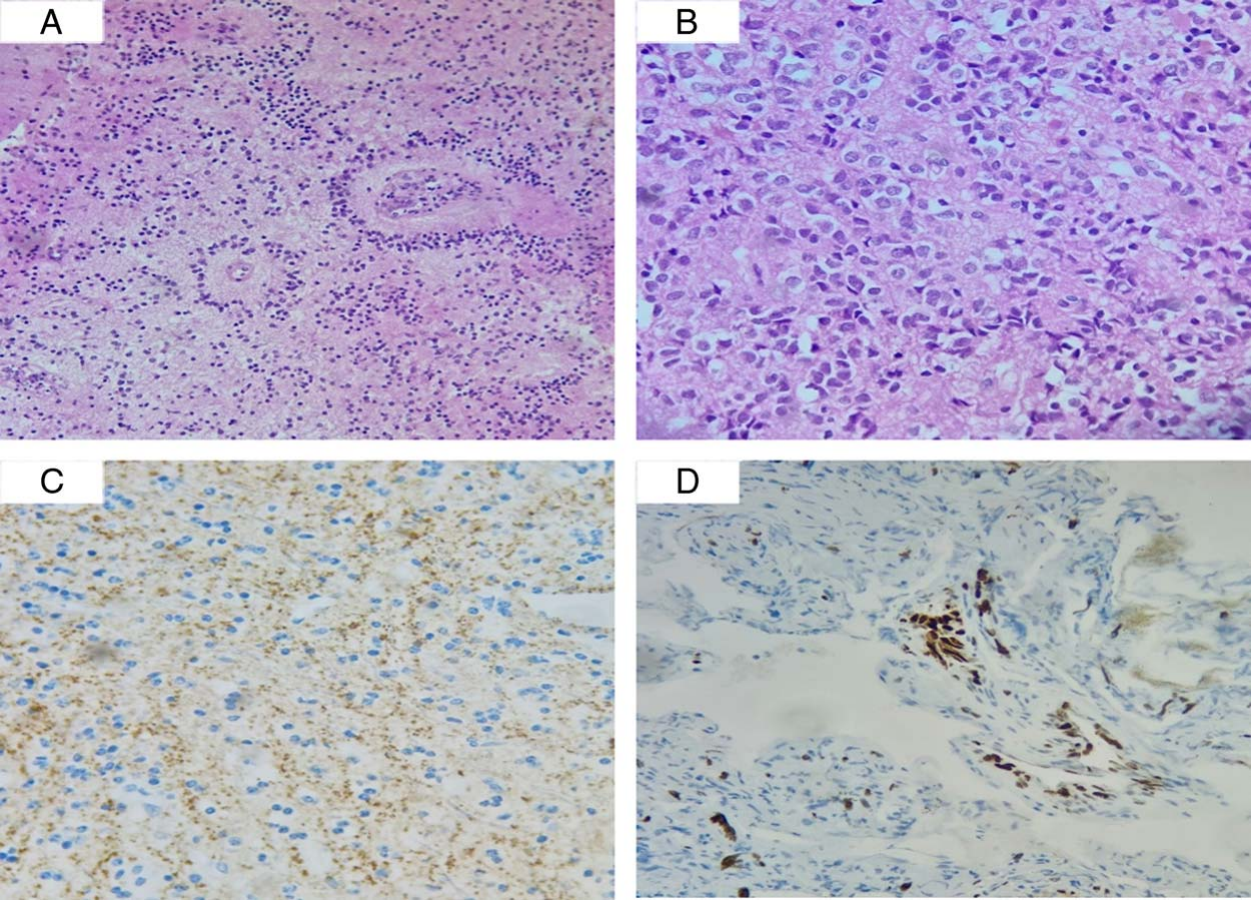
(A, B) Hematoxyline and eosin stains; (C) synaptophysin immunostain; (D) KI67 immunostain.

A 13-year-old female patient presented to the emergency department complaining of recurrent attacks of severe headaches for 1-month duration. The headache was associated with dizziness, blurred vision, nausea, and vomiting. Despite the chronic mild headache, the patient asserted that she had never experienced pain similar to this before. The patient reported no personal and/or family history of cancer, any acute, repeated, or discontinued medications, any allergies, any genetic or psychosocial issues, and had a free past surgical history. There was no history of trauma. Upon admission, physical assessment and routine laboratory evaluation, including a complete blood count and metabolic panel, were unremarkable. The initial brain neuroimaging was performed with a nonenhanced computed tomography scan and revealed a hypodense mass located superior to the cerebellum and posterior to the midbrain. MRI showed a heterogeneous mass demonstrating low T1, high fluid-attenuated inversion recovery (FLAIR)/T2 signal, and no definite diffusion restriction measuring 3.5×2.5×3 cm, resulting in compression over the cerebral aqueduct, which appears effaced with no cerebrospinal fluid (CSF) flow through the occluded duct. MRI also revealed a moderate supratentorial ventricular dilatation with periventricular edema (Fig. 1, arrows). The pineal gland could not be clearly identified. A pineal gland tumor was suspected. MRI of the spine showed no metastatic lesions. The patient was admitted to the department of neurosurgery and underwent an endoscopic third ventriculostomy under general anesthesia. Five days later, microscopic occipital craniotomy via a supracerebellar approach for total resection of the penial gland lesion was performed. The procedure was performed by a consultant at the neurosurgery department at a private hospital. The resected lesion was sent for routine histopathology, which showed a tumor with moderate cellularity arranged in a pseudo-rosette and sheet patterns, consisting of cells that are comparatively uniform in appearance with a weak eosinophilic cytoplasm, oval nuclei, and granular chromatin. The mitotic index was low. Immunohistochemical staining showed positivity for synaptophysin and KI67 (Fig. 2). In light of these findings, a PPTID was diagnosed. Postoperative MRI showed no residues of enhancing lesions and total relief of the obstructive hydrocephalus. The patient reported significant improvement in headache, nausea, vomiting, dizziness, and visual disturbances. She was started on a radiotherapy regimen as well as platinum plus etoposide chemotherapy. The patient adhered to and tolerated the provided advice, avoiding vigorous exercise and heavy lifting. She also had a good tolerance to chemotherapy agents and was followed up for 2 months without any reported adverse events or complications.

## Discussion

Melatonin is made by the pineal gland, a tiny, pinecone-shaped midline circumventricular structure. It develops embryologically from an ependymal outpouching and is situated posterior to the third ventricle. Less than 1% of intracranial neoplasms are pineal area tumors, making them uncommon^9^. As a result of the mass effect on neighboring structures, they frequently exhibit symptoms. CSF blockage and hydrocephalus are caused by compression of the cerebral aqueduct. Diplopia and Parinaud syndrome are brought on by tectum involvement. Nystagmus and ataxia may be caused by larger lesions that affect the cerebellum and its peduncles.

Neoplasms of the pineal gland include pineocytomas, pineoblastomas, PPTID, and papillary tumors of the pineal area. Desmoplastic myxoid tumor of the pineal area, SMARCB1-mutant (R), a rare SMARCB1-mutant tumor devoid of histological indications of malignancy, has been added to WHO CNS5 (The fifth edition of the WHO Classification of Tumors of the Central Nervous System). Pineal tumor behavior is still a subject of much debate, and histological grading standards for PPTID, papillary tumors of the pineal area, and desmoplastic myxoid tumor, SMARCB1-mutant have not yet been established^10^.

Neoplasms in the pineal area can develop from parenchymal pineal cells, leftover stem cells, or surrounding glia. Pineal parenchymal tumors, which make up about 27% of tumors in the pineal area, are so named because they develop from these cells^11^. Pineocytomas are more prevalent in females and adults, respectively. Pineocytomas may spread locally; however, this seldom happens^4^. They only undergo surgical treatment, and the prognosis is typically very good^12^,^13^. Poorly differentiated pineoblastomas are at the other end of the range from pineal parenchymal tumors. These extremely aggressive tumors have a 25–33% metastasis rate at diagnosis, which puts them at high risk for craniospinal metastases. The majority of pineoblastomas occur in youngsters. The prognosis is inversely correlated with the age of diagnosis, and 5-year survival is only 15% for children diagnosed at age 5 or under compared to 57% for those diagnosed at age 5 or older. Surgery, craniospinal radiation, and multimodal chemotherapy, with the possibility of myeloablative chemotherapy with stem cell rescue, are all used to treat these poorly differentiated neoplasms^13^,^14^.

Long known as malignant pineocytomas, pineocytomas with anaplasia, and atypical pineocytomas, these pineal parenchymal tumors are intermediately malignant between pineocytomas and pineoblastomas^6^. The WHO first acknowledged PPTID in 2000 after the term was coined in the 1990s^15^. Although formal histologic grading criteria have not yet been defined, PPTIDs are typically regarded as grade 2 or 3 tumors. There have been some suggested methods for differentiating between PPTIDs with lower and higher risks. One such system^3^ makes use of mitoses and antineurofilament staining, where high-grade PPTID is characterized by rare or absent antineurofilament staining or by six or more mitoses per high-power field, whereas low-grade tumors are characterized by positive neurofilament staining with fewer than six mitoses.

The clinical manifestation of a PPTID seems to be similar to that of other lesions in the pineal region. Diplopia and headache are the most common symptoms. Parinaud’s syndrome (abnormal vertical gaze as a result of tectal plate compression) is another frequent finding. If large enough, PPTID can cause hydrocephalus; obstructive (triventricular, noncommunicating) hydrocephalus, due to obstruction of the Sylvian aqueduct by the tumor, is the main underlying mechanism of the presenting symptoms in these patients^16^. Obstruction due to compression or neoplastic infiltration can be acute, subacute, or late (chronic)^17^. The commonest (∼90%)^18^ presenting symptoms of pineal space-occupying lesions are, therefore, those of raised intracranial pressure, presenting as mainly new and/or severe headache, occasionally chronic and/or unilaterally located, which improves at rest. Headache has been reported in up to 100% of patients with pineal meningiomas^16^.

For pineocytomas, surgery is frequently curative, but for pineoblastomas, severe chemotherapy (and frequently stem cell transplantation) is necessary. There is no agreement on how to treat PPTID, and many treatment paradigms that fall somewhere between these two extremes of aggression have been observed. The choice to utilize chemotherapy and radiation must be decided following the maximum safe surgical resection. The extent of the resection as well as the presence of spinal or CSF metastases, frequently play a role in this choice. The probability of craniospinal recurrence is intended to be decreased by systemic chemotherapy and more thorough ventricular radiation regimens. On the other hand, radiation and chemotherapy’s long-term harmful effects must be taken into account. These factors are weighed against the highly varied survival and recurrence risk associated with PPTID. In comparison to high-grade PPTID, low-grade PPTID has an estimated 5-year overall survival rate of 74%^4^.

Fifteen individuals first received surgical resection in Helsinki, Finland^19^; three of them had additional local radiotherapy since they were subtotal resections. Radiation therapy was only administered to patients on whom a complete gross resection was accomplished if the illness reappeared or if histology warranted it (pineoblastoma features or elevated MIB-1 index). No chemotherapy was administered.

After conducting a thorough analysis of 29 studies involving 127 patients with PPTID, Mallick *et al*.^20^ developed an alternative protocol in which, following the maximum safe resection, the choice of local radiation, craniospinal radiation, and surveillance is based on the presence of spinal metastases or CSF positivity as well as the degree of resection. Mallick *et al*. suggest surveillance with radiation saved for recurrence for large complete resections without spinal and/or CSF dissemination. Local radiation is pursued in situations of subtotal resections without spinal and/or CSF dissemination. Mallick *et al*.^20^ recommend craniospinal radiation followed by chemotherapy if there is an indication of spinal and/or CSF dissemination. The combined ventricular radiation and temozolomide regimen was effectively used in the management of PPTID^21^.

## Conclusion

We present the case of a 13-year-old female patient who had a big PPTID that was blocking her ventricular system and causing obstructive hydrocephalus. Physicians will be reminded of its prevalence and presentation by this case. One should carefully investigate a headache as the early symptom of many illnesses and rule out any other potential causes. This would therefore enable us to create a management structure for such a very unusual malignancy.

## Ethical approval

Our institution has exempted this study from ethical review.

## Patient consent

Written informed consent was obtained from the patient and family for the publication of this case report and accompanying images. A copy of the written consent is available for review by the Editor-in-Chief of this journal on request.

## Sources of funding

None.

## Author contribution

O.N.S. and A.W.M.J.: writing the manuscript; H.O., O.N.S., T.S., and M.A.: imaging description; O.N.S., N.S., Y.A., A.K., H.D., and S.H.: reviewing and editing the manuscript.

## Conflicts of interest disclosure

There are no conflicts of interest.

## Research registration unique identifying number (UIN)

1. Name of the registry: none.
2. Unique identifying number or registration ID: none.
3. Hyperlink to your specific registration (must be publicly accessible and will be checked): none.

## Guarantor

Oadi N. Shrateh.

## Provenance and peer review

Not commissioned, externally peer-reviewed.
